# On the enrichment of time series with textual data for forecasting agricultural commodity prices

**DOI:** 10.1016/j.mex.2022.101758

**Published:** 2022-06-17

**Authors:** Ivan José Reis Filho, Ricardo Marcondes Marcacini, Solange Oliveira Rezende

**Affiliations:** a State University of Minas Gerais - UEMG, Brazil; b University of São Paulo - USP, Brazil

**Keywords:** Text mining, Enriched series, Machine learning, Forecasting

## Abstract

Forecasting models in the financial market generally use quantitative time-series data. However, external factors can influence data in time-series, such as weather events, economic crises, and the foreign exchange market. This information is not explicit in the time-series and can influence the prediction of the variable values. Textual data can be a source of knowledge about external factors and is potentially helpful for time-series forecasting models. Some studies have presented text mining techniques to combine textual and time-series data. However, the existing representations have limitations, such as the curse of dimensionality and sparse data. This work investigates the finite use of domain-specific terms to investigate these problems by representing textual data with low dimensional space. We consider thirty-three keywords that are potentially important in the domain to enrich time-series using text mining techniques. Four regression models were applied to the representation proposed to predict the future daily price of corn and soybeans. The experimental setup considers a real market scenario, in which the daily sliding window strategy and step-forward forecast were used. The representation proposed has better accuracy in some forecasting scenarios. The results indicate that text data are a promising alternative for enriching time-series representations and reducing uncertainty forecasting models.

• We show an approach to enriching time-series using domain-specific terms;
• Representation proposed combines quantitative data with qualitative market factors;
• Regression Models to learn a forecasting function from enriched time-series.

## Graphical abstract

**Figure:**
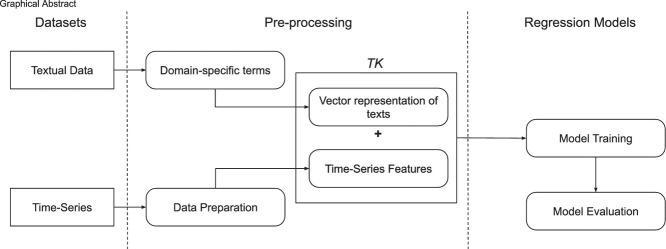

Specifications tablel

**Table:** 

Subject Area:	*Computer Science*
More specific subject area:	*Agricultural and Biological Sciences; Economics, Econometrics and Finance*
Method name:	*Time-Series Enriched with Domain-specific terms (TSED)*
Name and reference of original method:	N.A.
Resource availability:	https://github.com/ivanfilhoreis/tsed_commodities

## Introduction

Time-series data are commonly applicable for future price predictions in most applications and researches [1]. Traditionally, parametric and linear models have usually been explored for time-series forecasting [2], [3], [4], [5]. Introduced by [6], the ARIMA model has been one of the most popular approaches for time-series forecasting in different application domains. However, ARIMA-based models do not provide good predictions in more complex scenarios related to the financial market [7].

In order to overcome the limitations of the parametric models, non-parametric models have been proposed [8], [9], [10], [11], [12]. In particular, Machine Learning (ML) models have shown promising results with data-driven time-series forecasting models [13]. Artificial Neural Networks and Support Vector Regression are examples of non-parametric models that use only historical data to learn the stochastic dependency between the past and the future [14], [15], [16], [17]. Nevertheless, existing studies usually learn forecasting models exploring only trends and seasonality behavior of the historical time-series.

Regarding forecasts related to the financial market and commodities is a process challenge that involves stochastic and non-deterministic aspects. For example, the factors that influence the agricultural commodity include several variables that affect prices [18]. In addition to weather information, the factors can be categorized: i) Historical and recent market data; ii) Domestic demand and supply; iii) International demand and supply; iv) Macroeconomics; and v) Political factors. The first three factors are usually contained in time-series data. However, the last factors are more complex and subjective, generally available implicitly in texts extracted from news, social networks, and reports from different knowledge areas.

Text mining techniques have been used in studies to select text features and incorporate them into time-series [11], [19], [20]. The general idea is to extract a structured representation of the texts and associate them with price time-series. However, there are some limitations when applying vector space model representations of texts to prediction tasks. One of the main problems is the curse of dimensionality and sparse representations, as learning models with high-dimensional representations can be complex [21].

In order to research alternatives to these limitations, we consider a set of finite terms extracted from texts to enrich time-series with external factors available in textual information. In this work, models forecasting were used for regression tasks using three representations: Time-Series (TS), time-series Enriched with Domain-specific terms (TSED), and only Domain-Specific Terms (DST).

## Related works

Due to the variety of related works, the author divides them into three categories [22]: i) methods based only on technical information from time-series features, ii) methods based only on textual features, and iii) hybrid methods that combine textual features and technical information from time-series. This work scope is interested in hybrid methods, combining time-series and textual features to improve forecasting models. In this sense, Table 1 presents works related to different regression tasks. The column time-series (TS Domain) represents the temporal dependence and the domain of the data; the textual representation is the vector model used to enrich the predictive task; the training vs test presents the amplitude of the data in the experimental evaluation, and the Sliding Window (SW) represents the evaluation strategy used.

**Table 1:** Studies that combine technical information from time-series and textual features to improve the forecasting model.

Ref	TS domain	Textual rep.	Training vs Test	Algorithm	SW
[23]	AUD-USD daily prices	Bag-of-Words	Set. 2009 - Set. 2012 (60% train vs 40% test)	MLR, MLP	no
[20]	gold prices monthly	Clever Craft software	Jan. 1999 - Dec. 2005 vs. Jan. 2009 - Dec. 2009	ARIMA, ANN	no
[24]	daily oil price	TF-IDF	Nov. 2009 - Apr. 2012 vs. Mai 2012 - Jul. 2014	CNN, LDA	no
[25]	hourly taxi demand	GloVe embeddings	Jan. 2013 - Set. 2014 vs. Oct. 2014 - Jun 2016	DL-LSTM, DL-FC	no
[11]	average monthly prices of corn and soybeans	TF-IDF	Jan. 2014 - Feb. 2020	SVR	yes
[26]	average monthly prices of corn and soybeans	BERT	Jan. 2014 - Feb. 2020	SVR, LSTM	yes
[27]	S&P 500 index (monthly and yearly)	BERT	Jan. 2000 - Dec. 2019	ARIMA, LR, RF, FFNN, LSTM	yes
[28]	HSI daily closing price	LDA	Set. 2015 - Dec. 2020	Rolling Regression Model	yes

The works presented in Table 1 explore domain technical information to combine or analyze time-series observations. They are notably different in evaluating the test and training set, vector representation of texts, and semantic resources combined with time-series. The studies [11], [26] are our publications previous to this work. It is observed that the representation models and the prediction algorithms used are different. In addition, the data sources of time-series and texts are different in this work. In general, the hybrid models presented an increase in performance compared to time-series forecasting models. However, they have limitations, such as the curse of dimensionality and textual representations without considering important domain words. Thus, this work presents a representation of time-series enriched with specific domain characteristics for forecasting the daily prices of agricultural commodities.

## Methods

**Fig. 1:**
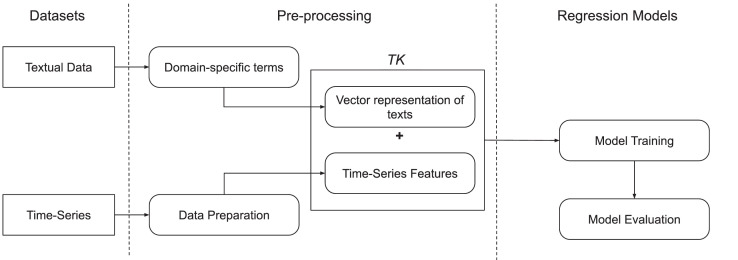
Conceptual Model of the TSED method.

This section presents the proposed method TSED, a representation of time-series combined with features extracted from a vector representation of texts. Fig. 1 illustrates the steps performed in the method.

### Pre-processing

A time-series S of size m is defined as an ordered sequence of observations, i.e., $S=(s_{1},s_{2},\ldots ,s_{m})$, where $s_{t}\in R^{d}$ represents an observation s at time t with d features. In the learning stage of a forecasting model, we consider different sizes u extracted from the time-series S, process called cross validation for times-series (Fig. 3). Thus, each step of forecasting we define a sequences $S_{u}=\left(s_{1},\ldots ,s_{u}\right)$, where u indicates the time period of the last observation of the time-series. Each sequence $S_{u}$ is associated with a forecast target value $y_{u+h}$, where h is the number of steps ahead, known as single-step ahead forecast with forecast horizon (h).

We present an approach to obtain a representation for the time-series, which considers the occurrence of specific words/terms (list of thirty-three words) in texts from the agricultural domain that can influence the time-series. Given a sequence $S_{u}$, we enrich this sequence with a vector representation of texts (BoW) that calculates the occurrence of domain words in the period $S_{u}$. First, we identify via time alignment all textual documents related to the sequence ($S_{u}$) and their respective representations in the vectorial space, as defined in Eq. (1) (Keywords Set).

(1) $$\begin{array}{cc} KS(1,u) & =Q(T,u)\\ & =\{B\left(d_{1}\right),B\left(d_{2}\right),\ldots ,B\left(d_{k}\right)\}\\ & =\{{\vec{v}}_{d1},{\vec{v}}_{d2},\ldots ,{\vec{v}}_{dk}\} \end{array}$$

where KS is a subset of texts (Q) with a text per day (T), and u indicates the number of days for the sequence. The vector representation (B) of each document $\left(d_{k}\right)$ is expressed as a vector ${\vec{v}}_{dk}$. The Term Frequency-Inverse Document Frequency (TF-IDF) was used to reflect how important a word is in the document collection. Then, the feature representation associated with the sequence is computed as an average vector from the document vectors, as defined in Eq. (2) (Keywords Features):

(2) $$KF\left(u,r\right)=\sum_{{\vec{v}}_{d}\in KS\left(u,r\right)}\frac{{\vec{v}}_{d}}{\left|KS\left(u,r\right)\right|}$$

The enriched representation is formed by the vector concatenation between the observations of the time-series and the Keywords features, TK(u)=S(u)⊕KF(u). Thus, we can use an enriched training set

(3) $$X=\{\left(TK_{u_{1}},y_{{\left(u+h\right)}_{1}}\right),\left(TK_{u_{2}},y_{{\left(u+h\right)}_{2}}\right),\ldots ,\left(TK_{u_{n}},y_{{\left(u+h\right)}_{n}}\right)\}$$

into the regression models, as presented in the next section.

### Regression models

After obtaining combined representations of the time-series and texts, indicating more qualitative information from the domain, the process continues to obtain regression models. In this work, we consider that non-linear regression models are more appropriate due to the chaotic nature of the time-series that requires textual information to reduce uncertainty. In this sense, we explored the Histogram-based Gradient Boosting Regression Tree (HGBR), Support Vector Regression (SVR), Random Forest Regressor (RF), and Bagging Regressor (BR). These four models has obtains promising results in several time-series forecasting works [9,^10^,[Bibr bib0029][Bibr bib0030].

A model is presented to a non-linear SVR forecast function to estimate a time-series [31]. In this work, the optimization process is done by estimating the multipliers $\alpha_{j}$ and $\alpha_{j}^{*}$, which represents the minimized objective function Eq. (4).

(4) $$L\left(\alpha \right)=\frac{1}{2}\sum_{i=1}^{N}\sum_{j=1}^{N}\left(\alpha_{i}-\alpha_{i}^{*}\right)\left(\alpha_{j}-\alpha_{j}^{*}\right)K\left(TK_{i},TK_{j}\right)+\epsilon \sum_{i=1}^{N}\left(\alpha_{i}-\alpha_{i}^{*}\right)-\sum_{i=1}^{N}y_{i}\left(\alpha_{i}-\alpha_{i}^{*}\right)$$

subject to

$$\sum_{i=1}^{N}\left(\alpha_{n}-\alpha_{n}^{*}\right)=0;\forall n:0\leq \alpha_{n}\leq C;\forall n:0\leq \alpha_{n}^{*}\leq C$$

where K is the kernel, ϵ defines a margin of tolerance where there is no given penalty for forecasting errors; and C is a previously defined positive constant that controls the penalty for observations that exceed the ϵ margin; which also helps to avoid excessive overfitting. The most common kernels are Polynomial, RBF, and Sigmoid. In this work, we consider Kernel RBF to have obtained the best results in the initial experiments.

Histogram-based Gradient Boosting Regression (HGBR) is inspired by LightGBM [32] and is a technique for training faster decision trees used in the gradient boosting ensemble. Model HGBR can be interpreted as:

(5) $$F_{M}\left(TK\right)=F_{0}\left(TK\right)+\sum_{m=1}^{M}F_{m}\left(TK\right)$$

where $F_{m}$ is built on a stagewise fashion, and each F is (LightGBM) a decision tree that executes M times using TK attributes. Random Forrest is an algorithm that handles large volumes of data within a relatively short computation time [33].

Random Forests (RF) for regression are formed by growing trees depending on a random vector Θ such that the tree predictor h(TK,Θ). The output values are numerical, and we assume that the training set is independently drawn from the random vector Y,TK distribution. The mean-squared generalization error for any numerical predictor h(TK) is:

(6) $$E_{TK,Y}{\left(Y-h\left(\mathrm{TK}\right)\right)}^{2}$$

The random forest predictor is formed by taking the average over k of the trees $h(\mathrm{TK},\Theta_{k})$. We kept the recommended^1^ number of trees (k=100). In order to reduce the size of the model, we changed the maximum tree depth parameters to four.

The Bagging Regressor (BR) is an ensemble meta-estimator that fits base regressors each on random subsets of the original dataset and then aggregates their predictions (either by voting or by averaging) to form a final prediction [34]. Assume we have a procedure for using learning set to form a predictor $\varphi (TK,\Gamma_{k})$, were Γ is learning set $\left(y_{n},TK_{n}\right)$. So, BR can be defined as:

(7) $$\varphi_{B}\left(TK\right)=av_{B}\varphi \left(TK,\Gamma^{\left(B\right)}\right)$$

where $\Gamma^{\left(B\right)}$ is base estimator to fit on random subsets of the dataset TK, φ is predictor with repeated bootstrap samples, and av is average all predictors $\varphi (TK,\varphi^{\left(B\right)})$. In this work, we consider the SVR as the basis of the estimator and the number of estimators (B=10). The presented regression models were used to investigate the effectiveness of incorporating domain-specific terms in time-series prediction tasks.

## Setup for experiment evaluation

This section presents evaluations of experiments using four regression models to compare the predictive performance of three representations: time-series (TS), time-series Enriched with Domain-specific Terms (TSED), and Domain Specific Terms only (DST). For assessing model performances and validity, the Mean Absolute Percentage Error (MAPE) statistical indicator was used.

**Fig. 2:**
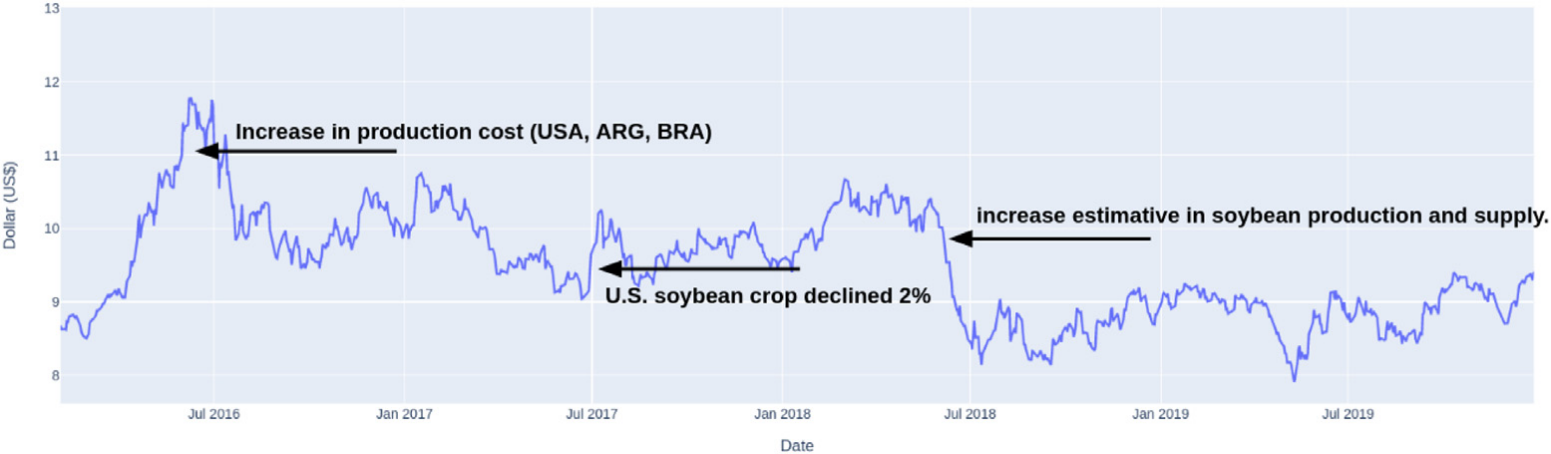
Soybean price series - Chicago of Board Trade (CBOT).

The time-series data source used in this experiment is from the Chicago Board of Trade (CBOT), available at CME^2^ Group’s website. Fig. 2 presents soybean prices series. We use the textual data extracted from the website Soybean & Corn Advisor^3^. Since 2009, the website has provided daily news and information on soybean and corn production related to the South American growth cycles, climate, infrastructure, land use, ethanol, and alternative fuel production.

Fig. 2 presents three examples of abrupt fluctuations in price series. By empirically analyzing the periods of price series that change a trend (high/low) or abrupt fluctuations in a few days, we observe a high occurrence of keywords in the news. Table 2 describes domain-specific keywords to enrich predictive tasks, the dataset period, the size of time-series datasets, and information about textual data.

**Table 2:** Overview of time-series and textual data used in experiment evaluation.

Commodity	Corn and Soybean
Period	2014-01-02 to 2020-12-30
Number of Days	1769
TS Attributes	Values (Open, Close, High, Low): CBOT
Number of News	1398
Domain-specific Keywords	crop, safrinha, losses, yield, estimate, disappoint, excellent, good, rains, planting, increase, decrease, price, reduction, sales, additional, complete, lower, low, more, progress, high, domestic, harvest, production, decline, cost, export, import, no news, record, large, growing

As shown in Table 2, the number of days in the time-series is different from the number of news items. Therefore, the term “no news” was considered for training and testing on days when there was no news on the site to maintain alignment between time series and texts.

To evaluate the proposed model, we use the Mean Absolute Percentage Error, presented in the Eq. (8).

(8) $$MAPE=\frac{1}{n}\sum_{i=1}^{n}\left|\frac{y_{i}-y_{i}^{\prime }}{y_{i}^{\prime }}\right|*100$$

where n is the number of testing samples, $y^{\prime }\left(i\right)$ is the actual value of each dataset, and y(i) is the forecasting value of the corresponding futures price data.

### Experiments and results

**Fig. 3:**
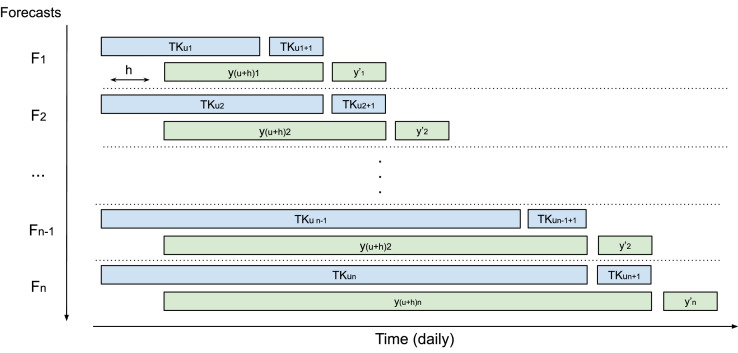
Cross-validation for time-series.

Considering the representation of the enriched time-series, expressed in Eq. (3), Fig. 3 illustrates how the method was applied in this work.

The cross-validation for time-series was used to evaluate the proposed model in the experimental evaluation. This strategy is mostly used in time-series forecasting contexts [35]. The first training step was performed with 30% of the data ($F_{1}$), and at each cross-validation iteration, a day is added to the training to predict the next step ahead. The variable $y^{\prime }$ in Eq. (8) represents the forecast of commodity prices h days ahead, and n represents approximately 1230 forecasts (daily) performed in the test stage. As presented in Section 3, four regression models were used to compare the predictive performance of representations. The Table 3 shows the set of hyperparameters used^1^.

**Table 3:** Hyperparameters used in regression models.

Model	Parameters
HGBR	default
SVR	Kernel RBF and gamma auto
RF	Depth = 4 and random state = 0
BR	base estimator SVR, estimator number = 10, random state = 0

**Fig. 4:**
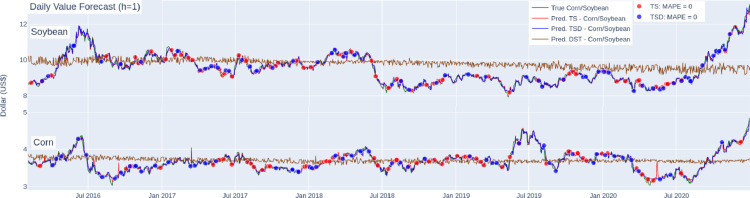
Predicted daily value for corn and soybeans with horizon (h=1).

After performing several structured experiments with different configurations, the hyperparameters of Table 3 were defined. Thus, Table 4 presents the MAPE values obtained in the forecast steps. In the experimental evaluation, five sizes of h were considered, that is, predicting one to five-time steps ahead. Values in bold are the smallest MAPE values of the regression model, and underlined are the smallest values of each representation (TS, TSED, DST). Fig. 4 shows the graph of the true and forecasted values of commodities with forecasting horizon h=1. The red and blue points represent the days when the forecast reached the MAPE equal to zero. The confidence level of new predictions can be measured by the average percentage error obtained in the results.

**Table 4:** Corn and Soybeans Results with forecast horizon (h).

Corn															
	TS	TSED	DST	TS	TSED	DST	TS	TSED	DST	TS	TSED	DST	TS	TSED	DST
Model	h = 1			h = 2			h = 3			h = 4			h = 5		
HGBR	**1,179**	1,186	7,554	**1,649**	1,687	7,578	**1,994**	2,021	7,579	**2,324**	2,341	7522	**2,589**	2,607	7,48
SVR (RBF)	**1,145**	1,240	6,056	**1,566**	1,632	6,036	**1,888**	1,953	6,015	**2,168**	2,220	5,993	**2,407**	2,450	5,985
RF	**1,167**	1,168	7,133	1,594	**1,593**	7,098	**1,920**	1,929	7,076	2,218	**2,215**	7,076	2,455	**2,454**	7,061
BR	**1,173**	1,263	6,788	**1,572**	1,64	6,789	**1,907**	1,954	6,763	**2,189**	2,222	6,73	**2,418**	2,455	6,692

Soybean															

HGBR	**0,982**	0,997	11,316	**1,375**	1,394	11,212	**1,714**	1,748	11,302	**1,987**	1,989	11,028	2,192	**2,157**	11,093
SVR (RBF)	1,022	**1,010**	7,611	1,382	**1,352**	7,560	1,696	**1,660**	7,568	1,947	**1,908**	7,528	2,147	**2,104**	7,506
RF	1,108	**1,107**	1,082	1,437	**1,434**	10,725	1,733	**1,728**	10,683	1,967	**1,964**	10,638	2,150	**2,142**	10,594
BR	**1,010**	1,027	7,807	1,369	**1,355**	7,791	1,659	**1,646**	7,772	1,906	**1,886**	7,727	2,104	**2,072**	7,672

According to the results presented in Table 4, the corn price forecast considering the TS representation, obtained the lowest MAPE values (values in bold) in almost all configurations (h). For example, analyzing the results of h=1, the SVR model with the TS representation had the lowest MAPE value with 1,145%, the RF had the lowest value for the TSED representation with 1,168%, and the SVR model had the lowest MAPE for DST representation with 6,056%. This pattern of the lowest MAPE value of the regression models for each representation is repeated for other forecast horizons (h).

Analyzing the results of the soybean price forecast in Table 4, the HGBR model obtained the lowest MAPE value for the TS and TSED representations for h=1, with values 0.982% and 0.997%, respectively. This pattern of the lowest MAPE value of the forecast models for each representation is not repeated for other forecast horizons h. However, the SVR model obtained the lowest MAPE values for the DST representation in all h horizons, with values 7,611%, 7,560%, 7,568%, 7,528% and 7,506%, respectively.

### Discussion

As presented in the experiments and results section (Fig. 4), DST representation predictions obtained an average of the price series. Thus, in this discussion, we will focus on analyzing the results of the TS and TSED representations that performed best (ie, results obtained from the underlined values of Table 4). In addition, Table 5 shows the number of days the representations had a lower MAPE value than the others.

**Table 5:** Comparison of the performance of representations in number of forecasts.

Corn					
Representions	h = 1	h = 2	h = 3	h = 4	h = 5
TS	547	570	545	570	489
TSED	418	455	466	480	441
TS = TSED	272	210	222	181	299
TS (MAPE 0%)	69	48	42	38	33
TSED (MAPE 0%)	57	48	50	30	28

Soybean					

TS	586	584	586	582	587
TSED	526	507	536	554	578
TS = TSED	125	144	111	95	64
TS (MAPE 0%)	67	52	44	40	41
TSED (MAPE 0%)	60	48	43	43	46

Analyzing the results of corn in Table 5, TS representation obtained 547 predictions in which the MAPE value was lower than the TSED, 418 predictions in which the TSED obtained a better result compared to the TS, and 272 in which both representations obtained equal values for the horizon (h=1). During the test phase, some predictions obtained the MAPE value equal to zero (0%), represented by dots (red and blue) in Fig. 4. In this case, TS and TSED representations obtained 69 and 57 very accurate predictions, respectively. The best performance of TS about TSED is repeated with a 16,7% superiority average in all forecast horizons (h).

The results of the soybean price forecast in Table 5 are similar to the corn results, where the TS representation obtained a more significant number of daily forecasts in all forecast horizons h. However, the superiority of TS over TSED is lower, with an average value of 7.6%. On the other hand, the number of predictions in which the TSED MAPE values were equal to the TS obtained a lower number.

We investigated the frequency of terms extracted from the texts and included in the time-series regarding the forecast days with a MAPE error equal to zero. The proposed representation performed well on days with abrupt intraday fluctuations in the price series. The Table 6 presents examples for h=1, where the date represents the day of publication of the news/headline and data prediction; the values in percentage represent the intraday oscillation; and the frequency that domain words occur in the news.

**Table 6:** News published in the previous days in which the price series showed abnormal fluctuations.

Corn				
Data	Headline	Prediction	Intraday	Keywords occurrence (News)
2020/01/30	Brazil to be a Major Exporter of Food to India in the Coming Years.	2020/01/31	1,05%	corn(1), export(3), increase(1), production(4)
2018/07/19	Brazilians may be missing Selling Opportunity due to Freight Dispute.	2018/07/20	-1,40%	additional(2), corn(1), cost(5), crop(6), estimate(2), harvest(1), high(4), import(1), increase(4), large(3), planting(1), production(1), rains(3), record(2)
2018/05/23	Initial Impact of Truck Strike on Brazilian Agriculture Sector.	2018/05/24	-1,47%	corn(2), cost(1), crop(1), domestic(1), export(10), good(1), harvest(1), high(2), increase(2), large(3), price(2), production(4), rains(7), record(2), safrinha(1)

Soybean				

2020/11/09	Brazil Importing U.S. Soybeans.	2020/11/10	3,24%	additional(3), domestic(3), export(2), harvest(2), high(1), import(7), large(2), planting(1), price(1), rains(1), record(2), sales(2), soybean(18)
2020/10/14	Full-Season Corn in Southern Brazil 39% Planted, About Average.	2020/10/15	-1,22	additional(1), crop(7), domestic(1), estimate(6), good(1), growing(3), harvest(2), high(3), increase(2), planting(13), price(4), production(3), rains(2), record(3), reduction(1), safrinha(11), soybean(3),
2017/02/07	Brazilian Government Announces Upgrade of Port of Santos.	2017/02/08	-0,84	complete(1), cost(1), export(4), good(1), import(4), increase(1), large(1), low(1), production(1), record(1), soybean(1)

According to the data presented in Table 6, the words corn, export, increase, and production have frequencies of 1, 3, 1, and 4, respectively, in the news published on 01/30/2020. Therefore, these words were used as resources in the TSED vector representation for a corn price forecast on 01/31/2020. The Term Frequency - Inverse Document Frequency (TF-IDF) measure was used to measure the importance of the word about text documents. The TF-IDF value is a weighting factor that increases proportionately as the number of occurrences in a document increases. Thus, words with high frequency in the texts had higher values, and words with little occurrence had lower values in the TSED representation. However, the TSED representation is based on independent words and does not express word relationships, text syntax, or semantics.

We also investigated the performance of the price forecast for the TS representation on the dates mentioned in Table 6. The TS representation did not perform well for the mentioned days. Furthermore, on the days when the TS representation performed better than the TSED, three situations often occurred: i) there was no news published on the dates; ii) they did not have much frequency of domain keywords; iii) the news content did not accurately represent the domain of the application. Regarding the last two, representation models that consider the semantics, linguistic structure, and context of texts can be proposed to mitigate this limitation, such as neural language models.

## Conclusion

Existing models have demonstrated a gained accuracy in predicting time-series. However, many studies do not consider external factors like market sentiment, politics, and other aspects. This work presented a time-series representation model enriched by Domain-Specific Expressions (TSED) to investigate these limitations. The proposed model was built from the matrix attribute-value representation, concatenated with the time-series, and applied in four regression models. Experimental results have demonstrated that ST representations perform better in most configurations. However, the TSED representation in some scenarios had better predictions than the TS.

In general, time-series representation models that consider textual information will hardly perform better at all prediction stages. However, the proposed model can be an alternative to help predict abrupt oscillations in time-series. Furthermore, enriched representations can contribute to the explicability of predictive models (black box). Future work can be carried out to extract more details from the texts, such as named entities, causal relationships, and techniques that consider semantic aspects to enrich the time-series. These techniques can help predict abrupt changes in time-series and explain predictive models.

## Declaration of Competing Interest

The authors declare that they have no known competing interests or personal relationships that could have appeared to influence the work reported in this paper.

## Footnotes

1 Number recommended by scikit-learn.org.

2 http://www.cmegroup.com/.

3 http://www.soybeansandcorn.com.
